# Supplementary material for: A versatile soluble siglec scaffold for sensitive and quantitative detection of glycan ligands
Source: Nat Commun. 2020 Oct 9;11:5091. doi: 10.1038/s41467-020-18907-6 (PMC7547722; doi:10.1038/s41467-020-18907-6)
Supplement: Supplementary file 3 — Reporting Summary [file 41467_2020_18907_MOESM3_ESM.pdf]

nature research

Corresponding author(s): Matthew S. Macauley

Last updated by author(s): Sep 14, 2020

Reporting Summary

Nature Research wishes to improve the reproducibility of the work that we publish. This form provides structure for consistency and transparency in reporting. For further information on Nature Research policies, see our Editorial Policies and the Editorial Policy Checklist.

Statistics

For all statistical analyses, confirm that the following items are present in the figure legend, table legend, main text, or Methods section.

n/a	Confirmed
☐	☒ The exact sample size (n) for each experimental group/condition, given as a discrete number and unit of measurement
☐	☒ A statement on whether measurements were taken from distinct samples or whether the same sample was measured repeatedly
☐	☒ The statistical test(s) used AND whether they are one- or two-sided<br>*Only common tests should be described solely by name; describe more complex techniques in the Methods section.*
☒	☐ A description of all covariates tested
☒	☐ A description of any assumptions or corrections, such as tests of normality and adjustment for multiple comparisons
☐	☒ A full description of the statistical parameters including central tendency (e.g. means) or other basic estimates (e.g. regression coefficient) AND variation (e.g. standard deviation) or associated estimates of uncertainty (e.g. confidence intervals)
☐	☒ For null hypothesis testing, the test statistic (e.g. F, t, r) with confidence intervals, effect sizes, degrees of freedom and P value noted<br>*Give P values as exact values whenever suitable.*
☒	☐ For Bayesian analysis, information on the choice of priors and Markov chain Monte Carlo settings
☒	☐ For hierarchical and complex designs, identification of the appropriate level for tests and full reporting of outcomes
☒	☐ Estimates of effect sizes (e.g. Cohen's d, Pearson's r), indicating how they were calculated

Our web collection on statistics for biologists contains articles on many of the points above.

Software and code

Policy information about availability of computer code

Data collection	BD FACSDivaTM software Version 8.0.1, Zeiss Zen Blue Edition
Data analysis	Graphpad Prism 8, FlowJo LLC. Version 9.9.6, Zeiss Zen Blue Edition Imaging Processing Software

For manuscripts utilizing custom algorithms or software that are central to the research but not yet described in published literature, software must be made available to editors and reviewers. We strongly encourage code deposition in a community repository (e.g. GitHub). See the Nature Research guidelines for submitting code & software for further information.

Data

Policy information about availability of data

All manuscripts must include a data availability statement. This statement should provide the following information, where applicable:

- Accession codes, unique identifiers, or web links for publicly available datasets
- A list of figures that have associated raw data
- A description of any restrictions on data availability

The authors declare that all data supporting the findings of this study are available within the paper and its supplementary information files. Please contact the corresponding authors (M.S.M.) for access of raw data, which is stored electronically, and will be made available upon reasonable requests. The coding sequence of Neuraminidase-A and Neuraminidase-S can be found in the GenBank; accession number: AY934539.2 for Neu-A and ABJ55283 for Neu-S.

Field-specific reporting

Please select the one below that is the best fit for your research. If you are not sure, read the appropriate sections before making your selection.

[x] Life sciences [ ] Behavioural & social sciences [ ] Ecological, evolutionary & environmental sciences

For a reference copy of the document with all sections, see nature.com/documents/nr-reporting-summary-flat.pdf

Life sciences study design

All studies must disclose on these points even when the disclosure is negative.

Sample size	For cell lines, sample size is 3 biological repetition. In human blood experiments the sample size is 3 biological repetitions for each sample. No sample size calculation was preformed, a sample size of 3 was chosen to make sure that there was no variance between biological replicates or samples. This was deemed sufficient as there was no significant variation between the replicates.
Data exclusions	No data was excluded from the study.
Replication	3 analytical replication done for cell line and human sample studies and all attempts at replications were successful.
Randomization	Randomization was not applicable for this study as only healthy controls were used and there was no comparison to other samples made.
Blinding	Blinding was not applicable to this study as only healthy controls were used and there was no comparison made to other samples.

Reporting for specific materials, systems and methods

We require information from authors about some types of materials, experimental systems and methods used in many studies. Here, indicate whether each material, system or method listed is relevant to your study. If you are not sure if a list item applies to your research, read the appropriate section before selecting a response.

Materials & experimental systems

n/a	Involved in the study	
[ ]	[x]	Antibodies
[ ]	[x]	Eukaryotic cell lines
[x]	[ ]	Palaeontology and archaeology
[x]	[ ]	Animals and other organisms
[ ]	[x]	Human research participants
[x]	[ ]	Clinical data
[x]	[ ]	Dual use research of concern

Methods

n/a	Involved in the study	
[x]	[ ]	ChIP-seq
[ ]	[x]	Flow cytometry
[x]	[ ]	MRI-based neuroimaging

Antibodies

Antibodies used	For human peripheral blood and spleen flow cytometry analysis, we used: Human TruStain FcX (Biolegend cat. no. 422302), hSig8 (PerCP/Cy5.5, clone 7C9, Biolegend cat. no. 347108), hCD123 (PE/Cy7, clone 6H6, Biolegend cat. no. 306010), hCD19 (PE, clone SJ25C1, Biolegend cat. no. 363004), hCD3 (FITC, BD Pharmingen cat. no. 555332), hCD14 (BV605, clone M5E2, BD Horizon cat. no. 564056), hCD15 (BUV395, clone HI98, BD Horizon cat. no. 563872), hCD56 (BV510, clone NCAM16.2, BD Horizon cat. no. 563041). For human peripheral blood samples, we additionally used hHLA-DR (APC-Cy7, clone L243, Biolegend cat. no. 307618). For other experiements, we additionally used: Strep-Tactin-HRP (HRP, IBA cat. no. 2-1501-001), and hIgG1-Fc (AF647, clone HP6017, Biolegend cat. no. 409320)
Validation	All the antibodies commercially purchased from these companies: link provided :1) https://www.biolegend.com/, 2) http://www.bdbiosciences.com/ca/applications/research/multicolor-flow/m/745795/horizon. Each of the antibody can be searched by their clone numbers on their respective website. In general all of the antibodies were quality control tested by immunofluorescent staining with flow cytometric analysis by the companies.

Eukaryotic cell lines

Policy information about cell lines

Cell line source(s)	HEK, Jurkat, A549, HuH7, U937, K562, BT549, MCF7, T47D, ZR-75-1, HS-578T, MDA-MB-231, MDA-MB-468
Authentication	All the parental cell lines were purchased from ATCC, cell lines were not authenticated within the lab.
Mycoplasma contamination	No Mycoplasma contamination detected (ABM Mycoplasma detection kit, Canada)

Commonly misidentified lines (See ICLAC register)	No commonly misidentified cell lines were used in the study.

Human research participants

Policy information about studies involving human research participants

Population characteristics	All participants used were randomly chosen and used as healthy controls. No previous medical information was asked for, age range was 20-40 years old, male and female participants and with no known genetic information about the participants was asked for or is known.
Recruitment	Participants were recruited on a volunteer basis by posters being distributed in public settings. Selection biases are not applicable as all samples were healthy controls and are not being compared to other groups.
Ethics oversight	University of Alberta Health Research Ethics Board - Biomedical Panel and the Institutional Review Board of the University of British Columbia (IRB#H17-01442)

Note that full information on the approval of the study protocol must also be provided in the manuscript.

Flow Cytometry

Plots

Confirm that:

- [x] The axis labels state the marker and fluorochrome used (e.g. CD4-FITC).
- [x] The axis scales are clearly visible. Include numbers along axes only for bottom left plot of group (a 'group' is an analysis of identical markers).
- [x] All plots are contour plots with outliers or pseudocolor plots.
- [x] A numerical value for number of cells or percentage (with statistics) is provided.

Methodology

Sample preparation	For human splenocytes, it was homogenized in media through a 40 µm filter (Corning) under sterile conditions. Samples were then centrifuged at 300 xg for 5 min. The resulting pellet from the spleen sample and whole human blood were treated with RBC lysis buffer (7 min) and centrifuged at 300 xg and the RBC lysis was repeated twice more until no RBC remained in the pellet. For cell line studies, cells were removed from the plate using 5 mM EDTA/PBS and centrifuged at 300 xg before staining.
Instrument	BD LSRFortessa TM X-20
Software	BD FACSDivaTM software V8.0.1
Cell population abundance	10000 for cell line studies and 100000-500000 for human blood and spleen studies
Gating strategy	All experiments are initially gated through SSC-A/FSC-A gating for live cells, further gated through SSC-W/FSC-A for singlet populations. For cell line studies, AF647 fluorescence was directly investigates, while for human peripheral blood and spleen samples, further gating was necessary in order to identify different subpopulations. Gating strategy is demonstrated in the Supplementary information and described in the methodology section of the manuscript.

- [x] Tick this box to confirm that a figure exemplifying the gating strategy is provided in the Supplementary Information.
